# Deletion of the Nucleotide Exchange Factor Vav3 Enhances Axonal Complexity and Synapse Formation but Tampers Activity of Hippocampal Neuronal Networks In Vitro

**DOI:** 10.3390/ijms21030856

**Published:** 2020-01-28

**Authors:** David Wegrzyn, Christine Wegrzyn, Kerry Tedford, Klaus-Dieter Fischer, Andreas Faissner

**Affiliations:** 1 Department of Cell Morphology and Molecular Neurobiology, Ruhr-University Bochum, Universitaetsstr. 150, Ruhr-University, D-44801 Bochum, Germany; David.Wegrzyn@rub.de (D.W.); Christine.Gottschling@rub.de (C.W.); 2 Institute of Biochemistry and Cell Biology, OVGU University of Magdeburg, Leipziger Str. 44, D-39120 Magdeburg, Germany; kerry.tedford@med.ovgu.de (K.T.); klaus.fischer@med.ovgu.de (K.-D.F.)

**Keywords:** axon growth, dendrite growth, guanine nucleotide exchange factor, Vav proteins, MAP2, multielectrode array (MEA), Rac1, RhoA family, Tau

## Abstract

Vav proteins activate GTPases of the RhoA subfamily that regulate the cytoskeleton and are involved in adhesion, migration, differentiation, polarity and the cell cycle. While the importance of RhoA GTPases for neuronal morphology is undisputed, their regulation is less well understood. In this perspective, we studied the consequences of the deletion of *Vav2*, *Vav3* and *Vav2* and *3* (*Vav2*^−/−^, *Vav3*^−/−^, *Vav2*^−/−^*/3*^−/−^) for the development of embryonic hippocampal neurons in vitro. Using an indirect co-culture system of hippocampal neurons with primary wild-type (WT) cortical astrocytes, we analysed axonal and dendritic parameters, structural synapse numbers and the spontaneous network activity via immunocytochemistry and multielectrode array analysis (MEA). Here, we observed a higher process complexity in *Vav3*^−/−^, but not in *Vav2*^−/−^ neurons after three and five days in vitro (DIV). Furthermore, an enhanced synapse formation was observed in *Vav3*^−/−^ after 14 days in culture. Remarkably, *Vav2*^−/−^*/3*^−/−^ double knockout neurons did not display synergistic effects. Interestingly, these differences were transient and compensated after a cultivation period of 21 days. Network analysis revealed a diminished number of spontaneously occurring action potentials in *Vav3*^−/−^ neurons after 21 DIV. Based on these results, it appears that Vav3 participates in key events of neuronal differentiation.

## 1. Introduction

The Vav subfamily of guanine nucleotide exchange factors (GEFs) is composed of the three members Vav1, Vav2 and Vav3 [1]. While the expression of *Vav1* is strictly limited to the hematopoietic system [2,3,4], *Vav2* and *Vav3* are also expressed in non-hematopoietic tissues and can be detected in the central nervous system (CNS) [5,6]. Vav proteins are regulated by receptor tyrosine kinases and activate their target proteins by catalysing the exchange of GDP by GTP [6,7]. In this way Vav proteins can activate members of the Rho GTPase family. Interestingly, GDP/GTP binding experiments could show that Vav proteins activate specifically RhoA, RhoG and Rac1 but not Cdc42 [8]. However, there are indications that Cdc42 is also influenced by Vav2 and Vav3 through an accumulation of phosphatidylinositol 3,4,5-trisphosphate [9].

Rho GTPases and their regulators participate in the development and plasticity of the CNS and are crucial for the organization of the cytoskeleton [10,11,12,13]. RhoA for example is involved in the semaphorin3a induced growth cone collapse of neurons [14]. In addition, experiments with dominant negative forms of Rac1 and Cdc42 showed a reduction of primary dendrites in multipolar neurons and basal dendrites in pyramidal neurons in rats [15]. Vav proteins act as activators of Rho GTPases and could exert an important regulatory function for the translation of extracellular signals into modifications of the cytoskeleton [11,16]. With regard to axonal growth, it was observed that Vav2/3-deficient retinal ganglion cells fail to collapse their growth cone after ephrin-A stimulation in vitro [17]. Furthermore, an induction gene trap screening revealed a correlation between the repulsive extracellular matrix protein tenascin-C and the upregulation of Vav3 in neural stem cells [18,19]. There are several studies indicating that a lack of Vav proteins leads to disturbances in the development of the CNS. The knockout of *Vav3* resulted in less branched Purkinje and granule cells in the cerebellum and led to motor deficiencies during the postnatal period. These deficits vanished in the adult stage [20]. Interestingly, the depletion of *Vav2* did not produce these abnormalities. Moreover, Vav3 is necessary for the correct axonal guidance of GABAergic neurons in the brainstem and thus responsible for physiologic cardiovascular and renal functions [21,22]. Recent studies revealed a connection between polymorphisms in the *VAV2* and the *VAV3* genes and cardiovascular risk factors [23]. Furthermore, a Japanese genome-wide association study could demonstrate that mutations in the *VAV3* gene seem to be associated with a higher risk for schizophrenia [24]. 

Experiments with in situ hybridizations demonstrated high mRNA levels of *Vav2* and *Vav3* in the hippocampus of mice (Allen Mouse Brain Atlas, http://www.brain-map.org) [25,26]. Along these lines, a comprehensive transcriptome analysis has demonstrated the expression of both *Vav2* and *Vav3* in CNS neurons [27]. In contrast *Vav1* is absent from neurons and glial cell types but abounds in mesodermally derived microglia [27]. Consistent with these observations, Vav3 has been documented in the growth cones of cultured hippocampal neurons after five days in vitro [22]. However, little is known about the function of Vav proteins for the early development and synaptogenesis of hippocampal neurons. Therefore, we cultured hippocampal neurons lacking *Vav2*, *Vav3* and *Vav2/3* in the presence of native cortical astrocytes in a co-culture setup. This system allows the cultivation of neuronal networks in completely defined medium and the subsequent analysis of structural synapses in vitro [28,29,30,31]. In this model, we first assessed the morphological differentiation of axons and dendrites using specific markers. In addition, multi-electrode array analysis [32] was performed to measure the spontaneous network activity of wild-type and *Vav3*^−/−^ neurons. 

## 2. Results

In view of the importance of small GTPases of the RhoA family for neuronal differentiation, we sought to determine the impact of the guanine nucleotide exchange factors Vav2 and Vav3 on neuronal morphology. We cultivated the neurons of one wild-type and three mutant (*Vav2*^−/−^, *Vav3*^−/−^ and *Vav2*^−/−^/3^−/−^) mouse lines in the presence of primary wild-type cortical astrocytes in order to guarantee identical culture conditions. Thus, any difference emerging in the neuronal monolayer of our cell culture system should be the direct expression of changes in the genotype caused by the deletion of the respective *Vav* genes. With this aim, hippocampal wild-type and knockout neurons were cultured for 3 or 5 DIV and immunostained with antibodies against the microtubule associated proteins MAP2 and Tau. While the expression of MAP2 is limited to the dendritic compartment [33], Tau proteins highly accumulate in the axon and take part in its stabilization [34]. Therefore, these markers are excellently suited to study the establishment of neuronal polarity

### 2.1. Ablation of Vav3 Increases Axonal Complexity and Dendritic Length after 3 and 5 DIV without Affecting the Number of Dendrites

**Figure 1:**
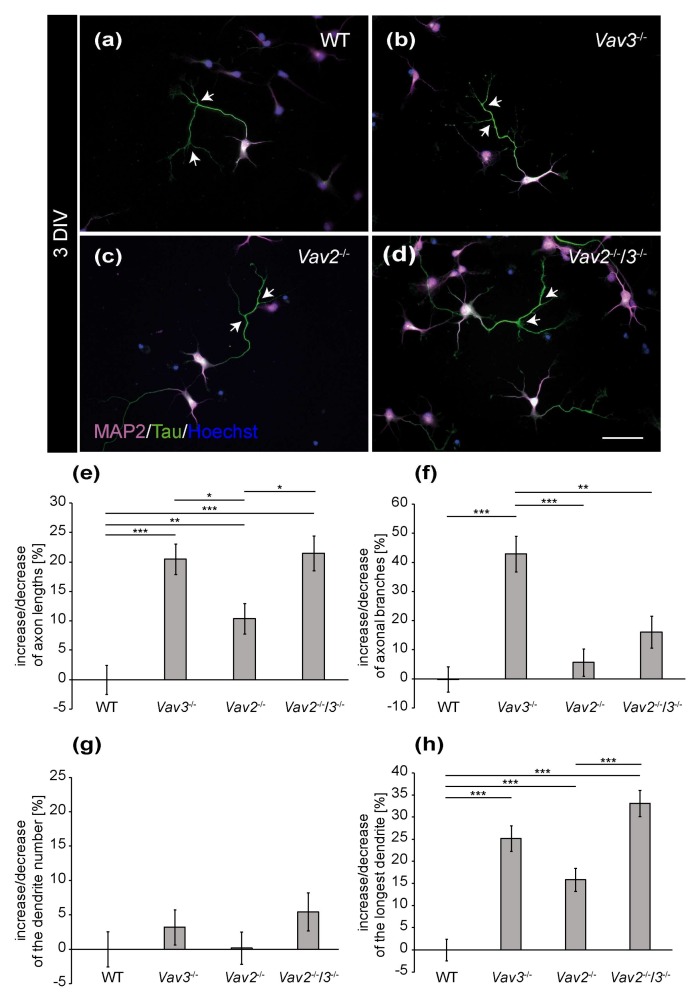
Immunocytochemical staining of MAP2 and Tau in hippocampal neurons after 3 DIV. (**a**–**d**) Hippocampal neurons of the different genotypes were cultured for three days and subsequently stained immunocytochemically with antibodies against MAP2 and Tau. While the expression of MAP2 (magenta) is restricted to the somata and dendrites, Tau (green) highly accumulates in the axon. Nuclei were visualized using bisbenzimide (blue) and axonal branches are marked by arrows. (**e**,**f**) The quantification of axonal parameters revealed that *Vav3*^−/−^ and *Vav2*^−/−^/*3*^−/−^ neurons developed significantly longer and more highly branched axons compared to the wild-type. (**g**,**h**) Analysis of the dendrites did not reveal any differences regarding the number but a significant increase of the longest dendrite in the knockout neurons. Statistics: five independent preparations (*N* = 5) were performed and 50–65 neurons (*n* = 50–65) were randomly chosen per condition, recorded and quantified concerning their axonal lengths and axonal branches, 30 neurons (*n* = 30) were quantified regarding the dendrite number and 45–50 neurons (*n* = 45–50) were used for the analysis of the longest dendrite. Data are shown as mean ± SEM (Kruskal–Wallis-test, *p* ≤ 0.05). Scale bar: 50 µm.

**Figure 2:**
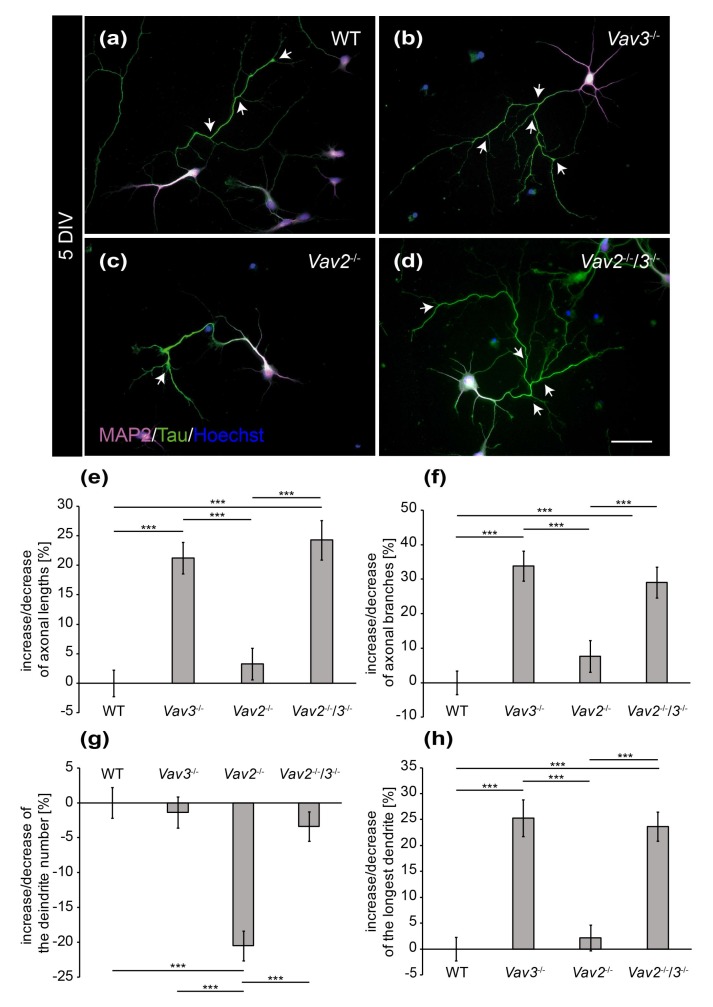
Immunocytochemical staining of MAP2 and Tau in hippocampal neurons after 5 DIV. (**a**–**d**) Hippocampal neurons derived from wild-type, *Vav2*^−/−^, *Vav3*^−/−^ and *Vav2*^−/−^/*3*^−/−^ mice were cultured for five days and immunostained with antibodies against the dendritic marker MAP2 (magenta) and the axonal marker Tau (green). Nuclei were stained with bisbenzimide (blue) and axonal branches marked with arrows. The representative images illustrate that *Vav3*^−/−^ and *Vav2*^−/−^/*3*^−/−^ neurons (**b**,**d**) form longer and more complex axons than wild-type and *Vav2*^−/−^ neurons (**a**,**c**); (**e**) *Vav3*^−/−^ and *Vav2*^−/−^/*3*^−/−^ neurons developed significantly longer axons than wild-type neurons. (**f**) The quantification of axon branches revealed a significant increase in *Vav3*^−/−^ and *Vav2*^−/−^/3^−/−^ neurons compared to wild-type and *Vav2*^−/−^ neurons; (**g**) *Vav2*^−/−^ neurons extended a reduced number of dendrites in comparison to all other conditions. (**h**) The average length of the longest dendrite was also significantly higher in *Vav3*^−/−^ and *Vav2*^−/−^/*3*^−/−^ neurons. Statistics: five independent preparations (*N* = 5) were performed and 50–65 neurons (*n* = 50–65) were randomly chosen per condition, recorded and quantified concerning their axonal lengths, 45–60 neurons (*n* = 45–60) were quantified for the axon branch analysis, 30 neurons (*n* = 30) were quantified regarding the dendrite number and 45–50 neurons (*n* = 45–50) were used for the analysis of the longest dendrite. Data are shown as mean ± SEM (Kruskal–Wallis-test, *p* ≤ 0.05). Scale bar: 50 µm.

The distribution of MAP2 and Tau allowed for a differential analysis of axonal and dendritic parameters of wild-type, *Vav2*^−/−^, *Vav3*^−/−^ and *Vav2*^−/−^/*3*^−/−^ neurons (Figure 1 and Figure 2). After a cultivation time of 3 days, neurons developed well-defined dendrites and axons (Figure 1a–d). Based on the immunocytochemical staining, slight differences in the complexity of *Vav3*^−/−^ and *Vav2*^−/−^/*3*^−/−^ neurons could be observed. Thus, dendrites and axons of *Vav3*^−/−^ and *Vav2*^−/−^/*3*^−/−^ neurons appeared longer and more complex compared with axons and dendrites of wild-type and *Vav2*^−/−^ neurons (Figure 1b,d). In contrast wild-type and *Vav2*^−/−^ neurons showed a similar morphology (Figure 1a,c).

To confirm these observations in a more detailed manner, different parameters were quantified and compared with each other (Figure 1e–h). First, the focus was set on the axonal compartment analysing the total number of branches and the average length. The analysis of the axonal length revealed interesting observations (Figure 1e). After 3 DIV, *Vav3*^−/−^ neurons developed the longest axons, which were significantly longer than axons of wild-type neurons (+20.45 ± 2.60%, *p* ≤ 0.001). Similarly, *Vav2*^−/−^/*3*^−/−^ neurons also showed elevated axonal lengths (+21.47 ± 2.95%; *p* ≤ 0.001) compared to wild-type. In addition, *Vav2*^−/−^ neurons were also significantly increased in axonal length but to less extent (+10.36 ± 2.61%, *p* = 0.004). Interestingly, *Vav3*^−/−^ neurons developed a significant increase in the total number of branches (+42.89 ± 6.08%, *p* ≤ 0.001, Figure 1f) compared to wild-type which was not significantly elevated in *Vav2*^−/−^/*3*^−/−^ neurons (+16.02 ± 5.50%, *p* = 0.320).

Next, we analysed dendritic parameters and determined the number of dendrites per neuron and the length of the longest dendrite (Figure 1g–h). Significant differences in the dendrite numbers could not be observed (Figure 1g). In this respect, neurons of all conditions formed about 5 dendrites per neuron. However, the analysis of the longest dendrite per neuron revealed that *Vav3*^−/−^ and *Vav2*^−/−^/*3*^−/−^ neurons formed significantly longer dendrites (*Vav3*^−/−^: +25.14 ± 2.86%, *p* ≤ 0.001; *Vav2*^−/−^/*3*^−/−^: +33.13 ± 2.98%, *p* ≤ 0.001, Figure 1h) than wild-type neurons. *Vav2*^−/−^ neurons developed slightly longer dendrites (+15.81 ± 2.62%, *p* ≤ 0.001) than wild-type neurons. However, the analysis of the sum of all dendrites per neuron did not reveal significant changes between all groups (see Supplementary Figure S1). The complete set of raw data can be found in the supplementary information (see Supplementary Table S1a).

To determine, whether these differences of fiber length are maintained in later stages of neuronal development, hippocampal neurons of all conditions were analysed after a cultivation period of 5 DIV (Figure 2). It appeared that both, the length and the complexity of axons and dendrites increased after 5 DIV (Figure 2a–d). Especially the axons of *Vav3*^−/−^ and *Vav2*^−/−^/*3*^−/−^ neurons attained a high degree of complexity, which was visibly higher compared to the wild-type and *Vav2*^−/−^ neurons (Figure 2b,d). 

The quantitative assessment of axonal and dendritic parameters after 5 DIV underlined these observations and revealed more extensive differences than the analysis after 3 DIV (Figure 2e–h). Thus, axonal lengths of all conditions were determined and compared with each other (Figure 2e). We observed that axons of *Vav3*^−/−^ neurons were significantly longer (+21.19 ± 2.66%, *p* ≤ 0.001) in comparison to the axons of wild-type neurons. Interestingly, analogous results were obtained when quantifying the lengths of *Vav2*^−/−^/*3*^−/−^ neurons (+24.27 ± 3.34%, *p* ≤ 0.001). This was an unexpected result because both Vav2 and Vav3 activate Rho GTPases and, consequently, might have the potential to compensate for each other. This was clearly not the case with regard to this parameter. In agreement with this result, *Vav2*^−/−^ neurons did not display altered axonal lengths compared with wild-type neurons. Next, analysis of the total number of branches revealed that *Vav3*^−/−^ neurons generated a significantly increased number of branches (+33.88 ± 4.35%, *p* ≤ 0.001, Figure 2f) compared to wild-type. In addition, *Vav2*^−/−^/*3*^−/−^ neurons also developed a clear rise of the total number of branches (+29.02 ± 4.45%, *p* ≤ 0.001). However, the deletion of both Vav-genes did not generate any cumulative effects regarding the complexity of axons. Consistent with this conclusion, *Vav2*^−/−^ neurons developed a comparable total number of axonal branches after 5 DIV as the wild-type. The analysis of the number of individual dendrites revealed no differences between wild-type, *Vav3*^−/−^ and *Vav2*^−/−^/*3*^−/−^ neurons (Figure 2g). However, it turned out that *Vav2*^−/−^ neurons developed a reduced number of dendrites (−20.48 ± 2.13%, *p* ≤ 0.001) compared with wild-type neurons. 

The measurement of dendritic lengths resulted in similar ratios as the analysis of the axonal lengths (Figure 1 and Figure 2h). In this respect, the longest dendrites of *Vav3*^−/−^ neurons reached the highest values and were significantly longer than the dendrites of wild-type neurons (+25.28 ± 3.57%, *p* ≤ 0.001). The dendrites of *Vav2*^−/−^/*3*^−/−^ neurons reached a similar length than the ones of *Vav3*^−/−^ neurons, surpassing the wild-type condition (+23.68 ± 2.81%, *p* ≤ 0.001). Here, the measurement of the total sum of all dendrites per neuron did not show significant changes (see Supplementary Figure S1b). The raw data concerning the axo-dendritic parameters after a cultivation time of five days in vitro can be found in the supplementary information (see Supplementary Table S2).

### 2.2. Vav3^−/−^ and Vav2^−/−^/3^−/−^ Neurons Form a Higher Number of Structural Synapses after 14 but Not after 21 DIV

**Figure 3:**
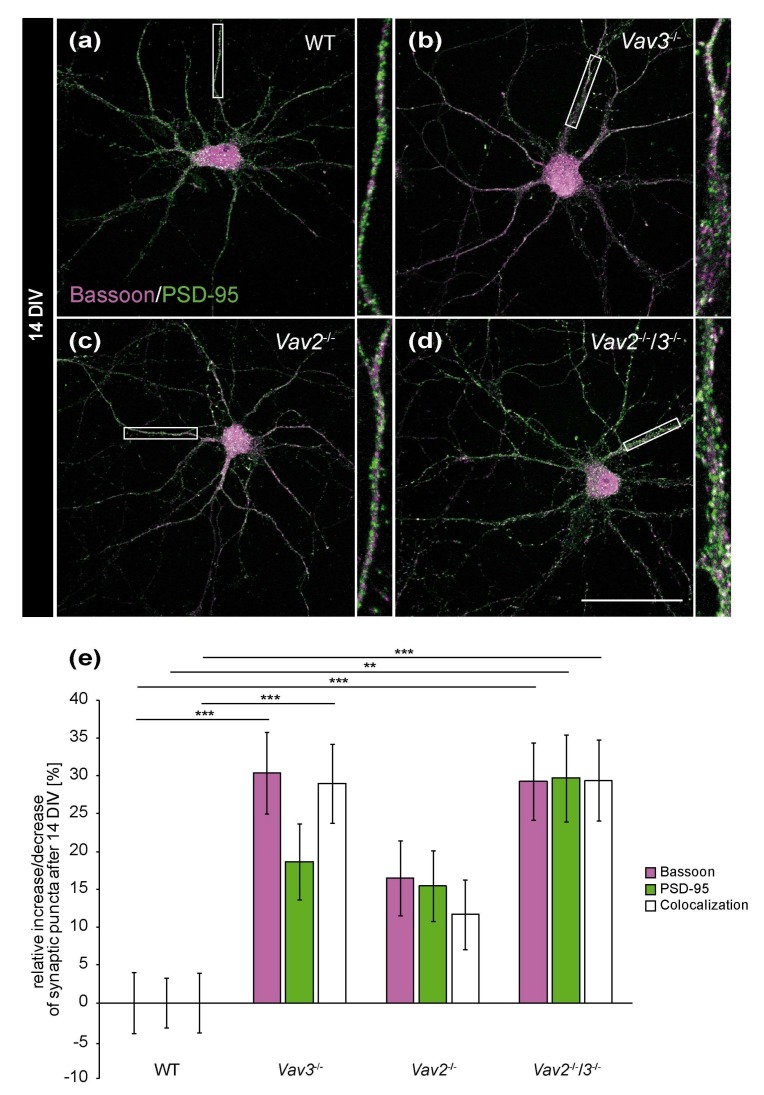
In vitro analysis of the synaptic puncta in hippocampal neurons derived from *Vav2*^−/−^, *Vav3*^−/−^ and *Vav2*^−/−^/*3*^−/−^ mice after 14 DIV. (**a**–**d**) Hippocampal neurons of all conditions were indirectly co-cultured with native cortical astrocytes, fixed after 14 DIV and stained immunocytochemically with antibodies against the presynaptic scaffold protein Bassoon (magenta) and the postsynaptic protein PSD-95 (green). A colocalization of both signals yielded in white puncta which were defined as indicators for structural synapses. (**e**) The quantification of synaptic puncta revealed a significant raise of presynaptic Bassoon puncta in *Vav3*^−/−^ and *Vav2*^−/−^/*3*^−/−^ neurons. The puncta for PSD-95 were only significantly increased in *Vav2*^−/−^/*3*^−/−^ neurons. Additionally, *Vav3*^−/−^ neurons formed 28.96 ± 0.39% and *Vav2*^−/−^/*3*^−/−^ neurons 29.38 ± 0.53% more colocalized signals than wild-type neurons after 14 DIV. Statistics: five independent experiments (*N* = 5) have been performed and 20 neurons (*n* = 20) of each condition were randomly recorded and quantified. Data are shown as mean ± SEM (Kruskal–Wallis-test, *p* ≤ 0.05) Scale bar: 50 µm.

To answer the question whether the enhanced axonal elongation and branching of *Vav3*^−/−^ and *Vav2*^−/−^/*3*^−/−^ neurons differentially influenced the formation of synapses, hippocampal neurons of wild-type, *Vav2*^−/−^, *Vav3*^−/−^ and *Vav2*^−/−^/*3*^−/−^ mice were co-cultured indirectly with cortical astrocytes for 14 and 21 days [29,35]. In order to analyse neuron-specific alterations caused by the lack of Vav proteins, exclusively primary cortical wild-type astrocytes were used for the co-cultures. Thereby, identical culture conditions were guaranteed for the different neuronal genotypes. Due to astrocytic factors released into the shared medium, it was possible to cultivate neuronal networks up to 21 days, as previously described [35]. At the end of the cultivation period, the cells were immunostained with antibodies against the presynaptic protein Bassoon and the postsynaptic protein PSD-95 to visualize and quantify structural synapses (Figure 3). The quantification of synaptic puncta after 14 DIV documented that *Vav3*^−/−^ and *Vav2*^−/−^/*3*^−/−^ neurons formed significantly more structural synapses than wild-type neurons, as certified by colocalized immunofluorescence signals (*Vav3*^−/−^: +28.96 ± 0.39%, *p* ≤ 0.001; *Vav2*^−/−^/*3*^−/−^: +29.38 ± 0.53%, *p* ≤ 0.001, Figure 3e). 

Concerning *Vav2*^−/−^ neurons, only a slight and not significant increase of colocalizations could be observed (+11.65 ± 0.46%, *p* = 0.285, Figure 3c,e). The selective quantification of the presynaptic scaffold protein Bassoon also revealed interesting differences. Here, a clear increase of Bassoon puncta was observed in the *Vav3*^−/−^ and *Vav2*^−/−^/*3*^−/−^ condition (*Vav3*^−/−^: +30.36 ± 0.54%, *p* ≤ 0.001; *Vav2*^−/−^/*3*^−/−^: 29.23 ± 0.51, *p* ≤ 0.001, Figure 3e). Additionally, an increase of PSD-95 puncta in *Vav2*^−/−^/*3*^−/−^ neurons was noted (+29.68 ± 0.56%, *p* = 0.002, Figure 3e). By comparison, the number of PSD-95 puncta was not significantly augmented in *Vav3*^−/−^ and *Vav2*^−/−^ neurons.

**Figure 4:**
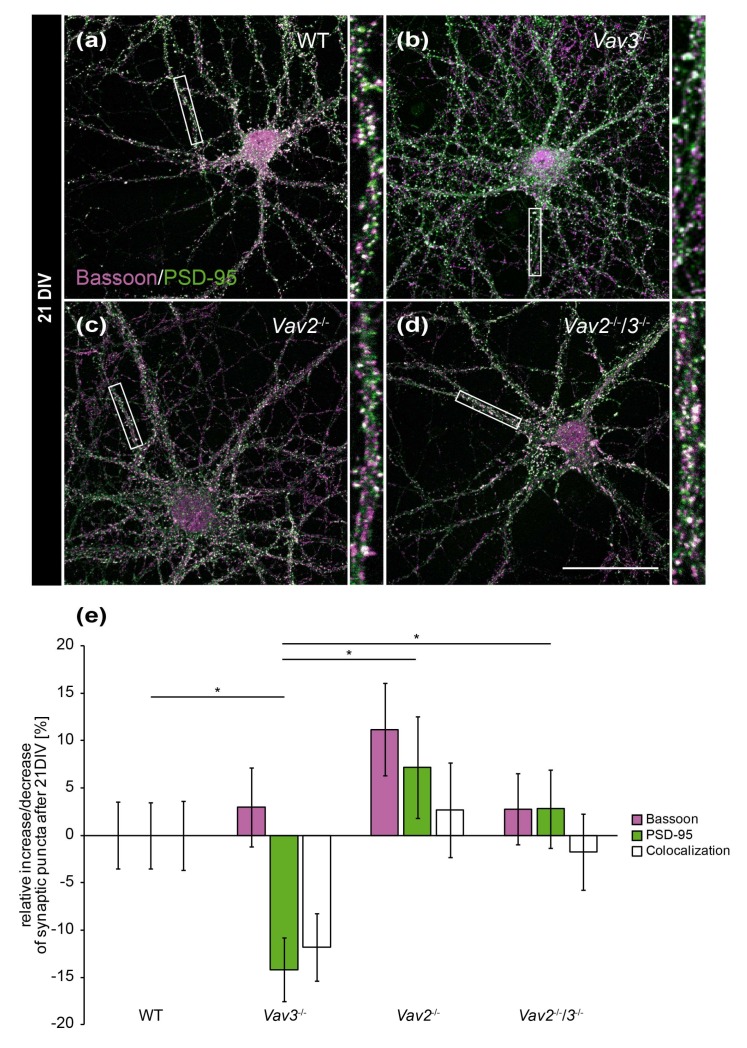
In vitro analysis of the synaptic puncta in hippocampal neurons derived from *Vav2*^−/−^, *Vav3*^−/−^ and *Vav2*^−/−^*/3*^−/−^ mice after 21 DIV. (**a**–**d**) Neurons derived from embryonic hippocampi of wild-type, *Vav2*^−/−^, *Vav3*^−/−^ and *Vav2*^−/−^*/3*^−/−^ mice were co-cultured with cortical astrocytes for 21 days and subsequently immunostained with antibodies against the presynaptic protein Bassoon (magenta) and the postsynaptic protein PSD-95 (green). The overlap of immunofluorescence resulted in white puncta, which are classified as indicative of structural synapses. (**e**) In contrast to the quantification after 14 days in vitro (Figure 3e), no significant changes could be detected between wild-type neurons and any knockout condition after 21 DIV. *Vav3*^−/−^ neurons exhibited a significant reduction of PSD-95 puncta in comparison to wild-type, *Vav2*^−/−^ and *Vav2*^−/−^*/3*^−/−^ neurons. Statistics: five independent experiments (*N* = 5) have been performed and 20 neurons (*n* = 20) of each condition were randomly recorded and quantified. Data are shown as mean ± SEM (Kruskal–Wallis-test, *p* ≤ 0.05). Scale bar: 50 µm. The raw data relating to the structural synapse analysis can be found in the supplementary information (see Supplementary Table S3).

Interestingly, these differences were not observable any more after 21 DIV (Figure 4e). This holds also true for colocalizations and for Bassoon puncta. Solely a slight reduction of PSD-95-positive puncta was detectable in *Vav3*^−/−^ neurons (−14.17 ± 0.37%, Figure 4e). This decrease was significant regarding the number of PSD-95 puncta of wild-type (*p* = 0.046), *Vav2*^−/−^ (*p* = 0.028) and *Vav2*^−/−^/*3*^−/−^ neurons (*p* = 0.022).

### 2.3. Vav3-Deficient Neurons Generated Neuronal Networks that Exhibit a Lower Spontaneous Network Activity and Reduced Synchronization after 21 DIV

**Figure 5:**
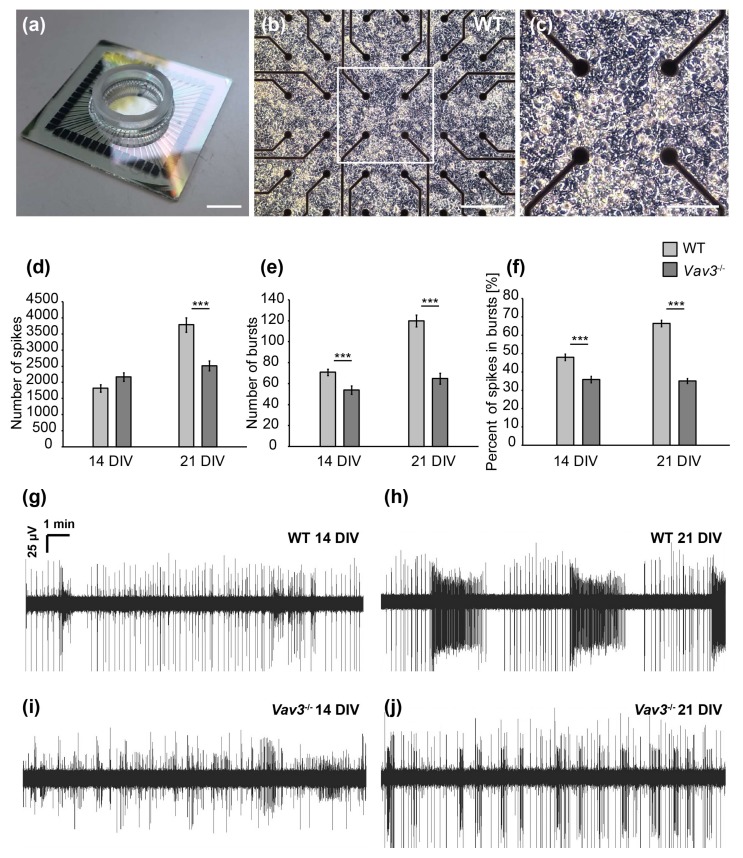
MEA analysis of spontaneous network activity after 14 and 21 DIV. (**a**) Illustration of a multielectrode array consisting of a centrally located electrode field limited by a glass ring (scale bar: 1000 µm). (**b**) Representative image of wild-type hippocampal neurons cultured on a poly-ethylenimine and laminin-1 coated MEA for 14 days in vitro. The electrode field is formed by 60 electrodes consisting of titanium nitride with a diameter of 30 µm. Action potentials that induce a change of potential in the immediate vicinity to the electrodes can be registered and recorded (scale bar: 100 µm). (**c**) Close-up of a wild-type cultured neuronal network after 14 days of culture. Neuronal cell soma as well as highly branched neurites can be seen (scale bar: 50 µm). (**d**) Analysis of single, spontaneously occurring action potentials (spikes) revealed no differences between wild-type and Vav3^−/−^ neurons after 14 DIV. However, the number of spikes was significantly reduced in neuronal networks lacking Vav3 in comparison to wild-type neurons after 21 days in vitro. (**e**) The organized activity of the network was qualified as a burst. Vav3^−/−^ neurons developed a significant decrease of bursts compared with wild-type neurons after 14 and 21 DIV. (**f**) Furthermore, the percentage of spikes occurring in well-organized bursts was quantified. Wild-type neurons showed a clear increase of spikes occurring in bursts from 14 to 21 DIV. Vav3^−/−^ neurons displayed a highly significant decrease in the proportion of spikes occurring in bursts compare to the wild-type condition. (**g**–**j**) Representative measurements over ten minutes of cultured hippocampal neurons derived from wild-type and Vav3^−/−^ mice after 14 and 21 days in vitro. Statistics: five independent experiments (*N* = 5) have been performed and the data of 60 electrodes (*n* = 60) was quantified per experiment. Data are shown as mean ± SEM (Mann–Whitney U-test, *p* ≤ 0.05). Scale bar: 100 µm.

Beyond the immunocytochemical analysis of structural synapses, MEA analysis was applied to examine functional changes of neuronal networks lacking Vav3 (Figure 5). To this end, wild-type and *Vav3*^−/−^ neurons derived from the hippocampus were cultured for 14 DIV and 21 DIV on MEAs followed by the measurement of the spontaneous network activity [32]. 

After 14 DIV, the number of spontaneously occurring actions potentials, also called spikes, was not significantly altered. Thus, 1817 ± 116.28 spikes were observed in wild-type and 2169 ± 131.13 spikes in *Vav3*^−/−^ neurons (*p* = 0.059, Figure 5d). After 21 DIV, an average spike number of 3785 ± 225.26 could be observed in wild-type neurons. Interestingly, *Vav3*^−/−^ networks produced an average spike number of 2515 ± 154.23 and were therefore significantly less active (*p* ≤ 0.001, Figure 5d). In addition, the coordinated network activity within a short time was analysed by measuring the so-called bursts [36,37]. After a cultivation time of 14 days, the number of bursts was significantly reduced in Vav3-deficient neuronal networks in comparison to wild-type neurons (wild-type: 71 ± 3.01; *Vav3*^−/−^: 54 ± 3.87, *p* ≤ 0.001, Figure 5e). After 21 days in vitro, 120 ± 5.62 bursts could be measured in wild-type neurons. The average number of bursts in *Vav3*^−/−^ neurons amounted to 65 ± 5.22 at that stage and were significantly reduced (*p* ≤ 0.001, Figure 5e). Last, the percentage of spikes occurring in well-organized bursts was analysed in order to investigate the maturation of neuronal networks in vitro (Figure 5f). After 14 days in vitro, wild-type neurons developed a proportion of 48.01 ± 1.8% spikes within bursts. After 21 days in culture, an elevation to a ratio of 66.46 ± 1.7% could be observed in wild-type neurons. Interestingly, the percentage of spikes occurring in bursts was significantly reduced in *Vav3*^−/−^ neurons after 14 (35.94 ± 1.76%) and 21 DIV (35.11 ± 1.41%) in comparison to the wild-type neurons (*p* ≤ 0.001).

In summary, the multielectrode array analysis revealed a significantly downregulated spontaneous network activity of embryonic hippocampal neurons derived from *Vav3*^−/−^ mice. While an increasing neuronal activity could be observed in wild-type neurons from DIV14 to DIV21, the activity of *Vav3*^−/−^ neurons stagnated after 14 days in vitro. The number of spikes, bursts and the percentual amount of spikes in bursts was significantly ameliorated in knockout cultures. 

## 3. Discussion 

In the present study, we investigated the role of Vav proteins for the development of neuronal polarity, synapse formation and spontaneous network activity in vitro. We found a significantly increased axonal complexity accompanied with an increased number of presynaptic terminals but reduced levels of activity in neuronal cultures derived from Vav3 lacking embryonic mice. Our observations indicate that in particular Vav3 has important regulatory functions concerning axonal development and synapse formation. In contrast, Vav2 appears to play only a minor role for the development of hippocampal neurons in vitro. 

The morphological analysis revealed that hippocampal neurons lacking Vav3, Vav2/3 but not Vav2 form longer axons with a higher level of complexity. Several studies have shown that Vav proteins are potential activators of Rho GTPase family members [7,8]. It has been reported that Vav3 strongly unfolds its nucleotide exchange activity with the targets RhoA and RhoG, whereas Rac1 is activated less efficiently, and Cdc42 not at all [6,8,38]. Interestingly, Vav3 has been found enriched in hippocampal synapses [39] and in hippocampal neuron axons after 5 days in vitro [22].

*Vav3*-deficient GABAergic neurons of the ventral medulla show deficits in target homing and synapse formation in the brainstem [22]. In the retino-geniculate projection, Vav proteins are required for the induced endocytosis of Eph-ephrin ligand receptor complexes [17]. Neuronal growth cones are specialized and chemo sensitive structures, which respond to extracellular signals with retraction or protrusion of filopodia [40]. Interestingly, Vav proteins activate RhoA, which is implicated in the formation of stress fibres and the collapse of axonal growth cones [14,41,42,43,44]. Rac1, another prominent member of the Rho GTPase family, participates in the collapsin and semaphorin induced growth cone collapse [45]. The lack of Vav3 may result in a reduced activity level of RhoA.

Therefore, axonal growth cones of *Vav3*^−/−^ neurons might be less responsive to repulsive extracellular signals. This may represent a possible explanation for the increase of axonal lengths and branching. The extracellular matrix protein tenascin-C exerts repulsive effects on developing axons of neurons [18,46]. Interestingly, a gene trap analysis performed on neural stem cells revealed that Vav3 message is suppressed by tenascin-C [19]. Based on earlier reports and our observations, we propose that Vav3 is an important regulator for intracellular rearrangements of the cytoskeleton, thereby playing an important regulatory role for axonal path finding and dendrite elongation in the developing CNS. 

Beside the increased axonal lengths, longer dendrites could be observed in *Vav3*^−/−^ and *Vav2*^−/−^/*3*^−/−^ neurons, foremost after 5 DIV. Vav3 seems required for the dendritic branching of Purkinje cells in the cerebellum [20]. The downstream targets of Vav proteins play a crucial role for the development of dendrites. The expression of a constitutively active form of RhoA resulted in an impaired dendritic growth in rat neurons [47]. Conversely, the dominant negative form of RhoA enhanced the development of dendrites in murine hippocampal neurons in vitro [48]. The activation of RhoA is often associated with adverse effects concerning dendrite development. Therefore, RhoA might be responsible for the limitation of dendritic growth. A reduced activity level of RhoA caused by the deletion of Vav2 and Vav3 may therefore entail a reduced inhibition of dendrite development. This could explain the increased dendritic lengths of *Vav3*^−/−^ and *Vav2*^−/−^*/3*^−/−^ neurons observed in our study. Rac1 represents another regulator of dendritic morphology that antagonizes the effects of RhoA. In cortical neurons, the expression of a dominant negative Rac1 caused a reduction of basal dendrites and dendritic spines in cortical neurons [15,47]. Conversely, expression of constitutively active Rac1 enacts extensive branching of Purkinje cells [49]. In our studies, we measured promotion of the dendrite lengths of *Vav3*^−/−^ and *Vav2*^−/−^*/3*^−/−^ neurons. Because Vav3 modulates primarily the activation of RhoA and to a much lesser extent of Rac1 [6], a reduced activation of RhoA consequent to the deletion of Vav3 could be an appealing explanation for the enhanced axonal and dendritic development. To prove this hypothesis, experiments with Rho activators would be interesting and should reveal whether the observed phenotype can be reversed. 

The morphological analysis of the dendritic complexity after a longer cultivation time represents an interesting theme. Because the neuronal networks in our assay system develop intricate fibre networks within two weeks, the tracing of dendrites to individual cells is not reliably possible. Therefore, we limited ourselves to brief cultivation periods, which resulted in the simplicity of formed dendrites. For these reasons, potential long-term effects regarding dendrite arborisation could not be addressed in our study. 

Our results indicate that *Vav3*^−/−^ and *Vav2*^−/−^*/3*^−/−^ neurons developed a higher number of structural synapses after 14 DIV. We suggest that augmented synaptogenesis arose on the one hand from the enhanced axonal growth and, on the other hand, from stimulated dendrite development. The increased number of axonal branches and lengths may translate into the rise of presynaptic terminals and eventually produce a higher number of co-localized puncta. In accordance with this assumption, more presynaptic Bassoon puncta were visible after 14 DIV. Along these lines, the expansion of dendrites probably enhances the target areas for the axonal growth cones. Interestingly, the Rho GTPases RhoA and Rac1 regulate the formation and maintenance of dendritic spines in hippocampal pyramidal neurons and in Purkinje cells [47,49,50,51]. For example, the inhibition of RhoA using the enzyme C3-transferase caused an abnormal increase of dendritic spines in hippocampal pyramidal neurons [50]. In contrast, a dominant negative form of Rac1 did not enact noteworthy differences in spine morphology [50]. As the Vav proteins impinge on both RhoA and Rac1, it is difficult to judge which of the potential pathways is implicated in synaptogenesis. After 21 DIV, all cultures attained a comparable number of synaptic puncta. This indicates that wild-type and *Vav2*^−/−^ neurons eventually reached the same level of complexity as Vav3^−/−^ and Vav2^−/−^/3^−/−^ neurons, however, on a protracted time scale. These observations highlight Vav3 as an important regulator of the initial neuronal development in vitro. Interestingly, cerebellar development is altered in *Vav3*^−/−^ mice. Behavioural experiments revealed deficits concerning motor coordination and gaiting during the early postnatal period of *Vav3*^−/−^ mice that ameliorated in older animals [20,52]. 

In our studies using MEAs, we could measure a decrease of spontaneous and of organized network activity in Vav3 deficient neurons. Little is known about the roles of Vav proteins and their downstream targets, RhoA and Rac1, concerning the activity of neuronal networks. Interestingly, some studies indicate an important role of Rho GTPases for neurosecretion [12]. Recent findings revealed that Rho GTPases are activated during synaptic transmission in the CA1 region of rat hippocampus [53]. Furthermore, the activation of RhoA and Rac1 by CNF1 leads to enhanced neurotransmission, synaptic plasticity and improved learning and memory in mice [54]. Conversely, the RhoA inhibitor C3 transferase decreased the presynaptic release of acetylcholine in *C. elegans* [55]. The reduced spontaneous network activity of *Vav3*^−/−^ neurons on MEAs could hence be explained by an impaired release of neurotransmitter. Although we observed an increased number of structural synapses after 14 DIV, the number of spontaneously occurring action potentials was reduced at this point in time. An increased number of structural synapses is not necessarily accompanied by an increase in electrophysiological activity and might be explained by the presence of so-called silent synapses. Silent synapses have been described in the developing hippocampus and are characterized by the lack of AMPA-receptors in the postsynaptic compartment, rendering them unable to mediate synaptic transmission under physiological conditions [56,57]. This type of synapses is supposedly detectable in immature synapses and might indicate an impairment of synaptic maturation in neurons lacking Vav3. Interestingly, a compromised glutamatergic synapse maturation and AMPA-receptor stability was observed when the Rho-GTPase-activating protein oligophrenin-1 was defective or decreased [58]. Several reports emphasize further links between mental retardation syndromes and alterations in small GTPases or GTPase regulating proteins [59,60]. 

While we observed differences in axonal and dendritic morphology as well as in synapse formation and network activity of *Vav3*^−/−^ and *Vav2*^−/−^*/3*^−/−^ neurons, *Vav2*^−/−^ neurons exhibited only slight or no visible modifications. Furthermore, the *Vav2*^−/−^*/3*^−/−^ neurons did not display evidence of cumulative effects and resembled in their morphology the *Vav3*^−/−^ neurons. This is indicative of a negligible role of Vav2 with regard to the development of axons and dendrites. On the other hand, this finding underlines that the genetic ablation of a *Vav* gene by itself is not sufficient to impact the cell biology of neurons. In this sense, the *Vav2^−/−^* can be considered a supplemental independent control for the *Vav3^−/−^* phenotype, beyond the wild-type. 

The sole phenomenon of significance was a reduced number of dendrites after 5 days in vitro. This phenotype of *Vav2*^−/−^ neurons vanished at later time points of cultivation. Analogous observations with respect to Vav2 were reported concerning the development of the cerebellum [20]. Alternatively, Vav2 and Vav3 might be expressed to different degrees in the CNS. In fact, in situ hybridizations revealed high expression levels of Vav3 message in particular in the hippocampus and the cerebellum but a generally lower expression of *Vav2* (Allen Mouse Brain Atlas, http://www.brain-map.org) [25,26]. Therefore, it is conceivable that *Vav3* compensates the knockout of *Vav2* whereas the opposite option is improbable. Of note, the Vav protein family forms a small subgroup of the “transforming oncogene of diffuse B-cell lymphoma” (DBL) family of GEFs that comprises more than 60 genes in the human [61]. Therefore, not only GEFs of the Vav protein family might compensate the knockout of *Vav2* but also GEFs of other protein families. The great number of GEFs poses a serious challenge for the identification of compensatory mechanisms and should be the aim of follow-up projects. 

According to current views, the expression level of small GTPases of the RhoA-family is not rate limiting for their respective activity. Rather, the activation state depends on the controlling GEFs, GAPs and GDIs in the cellular context [62]. In support of this notion it has been reported that the levels of activated Rac1 are reduced in immune cells of the Vav2 knockout mutant, while its protein levels are unchanged. We therefore conclude that the differences we observe in our study are not a consequence of varying levels of effectors [63,64].

In summary, our data revealed that primarily Vav3, but not Vav2, is involved in axon elongation and branching, in dendrite development and, finally, in synapse formation of hippocampal neurons in vitro. Future studies with transgenic knockout mice may focus on the behaviour of axonal growth cones in distinct extracellular microenvironments. In that regard, it will be of interest to examine whether Vav proteins regulate or tune the growth cone response to promoting or repulsive stimuli. In this context, the identification of GTPases controlled downstream of Vav represents an important objective which will require the development of tools to monitor GTPase activities in small cell populations. In the light of the current knowledge, members of the RhoA subfamily of GTPases represent attractive candidates. For example, current studies focus on the therapeutic inhibition of RhoA, which is correlated with an enhanced neuronal survival and axon regeneration after lesion [65,66,67,68,69]. Progress in this research field may help to develop new therapies and strategies to treat CNS lesions. 

## 4. Materials and Methods 

### 4.1. Animals

Wild-type SV129, *Vav2*, *Vav3* and *Vav2/3* knockout mice were used in accordance with the Society for Neuroscience and European Union guidelines, European Council Directive of September 22, 2010 (2010/63/EU), for care of laboratory animals and approved by the animal care committee of North Rhine-Westphalia, Germany, based at the LANUV (Landesamt für Umweltschutz, Naturschutz und Verbraucherschutz, Nordrhein-Westphalen, D-45659 Recklinghausen, Germany). The animal wellfare commissioner of Ruhr-University supervised the study. Animals of both sexes were used for the experiments and kept under standardized conditions with regulated humidity and 24 h dark/light circle. The supply with food and water was ensured *ad libitum*. Wild-type SV129 and *Vav3*^−/−^ mice originate from the mouse breed of the Department for Cell Morphology and Molecular Neurobiology of the Ruhr-University Bochum [70]. The *Vav2*^−/−^/*3*^−/−^ double knockout mouse line [71] was used to outbreed Vav2^−/−^ mice [72] and both lines were kept in the animal facility of the faculty of Biology and Biotechnology of Ruhr University.

### 4.2. Immunological Reagents

The following rabbit polyclonal antibodies were used: anti-bassoon (1:1000; RRID:AB_887697; Synaptic Systems), anti-MAP2 (1:300; RRID:AB_1840999; Sigma-Aldrich (by Merck KGaA), Darmstadt, Germany. Additionally, the monoclonal mouse antibody against postsynaptic-density protein 95 (PSD-95, 1:500; RRID:AB_94278; Merck Millipore, Darmstadt, Germany) and the monoclonal chicken antibody against Tau (1:300; RRID:AB_1310734; Abcam) were used. All secondary antibodies were obtained from Dianova. These were labelled with Cy3 (1:500) or AF488 (1:250) and showed specificity for the primary mouse, rabbit and chicken antibodies. The detection of cellular nuclei occurred with bisbenzimide (Hoechst 33258, 1:100.000; Sigma-Aldrich). 

### 4.3. Dissection and Cell Culture

To analyse the synaptogenesis, hippocampal neurons and cortical astrocytes were co-cultured indirectly [29,35,73]. For the cultivation of cortical astrocytes, postnatal mice (P1-P3, SV129) were decapitated. Afterwards, the cortices were extracted and freed from the meninges. The cortices were incubated in a digestion solution containing 30 U Papain (Worthington), 40 µg/mL µL DNase I (Worthington) and 0.24 mg/mL l-cysteine (Sigma-Aldrich) in DMEM (Gibco) for 1 h at 37 °C. Then the digestion solution was removed and replaced by astrocyte medium composed of DMEM (Gibco) with 10% *v*/*v* horse serum (Gibco) and 0.1% *v*/*v* gentamicin (Sigma-Aldrich). After stopping the process of digestion, the tissue was triturated mechanically and centrifuged for 5 min at 1000 rpm. The supernatant was discarded, and the cell pellet was resuspended in 1 mL fresh astrocyte medium. After this, 1 mL single-cell suspension was added to 9 mL of astrocyte medium in a T-75 culture flask (Sarstedt) pre-coated with 10 µg/mL poly-D-lysine (Sigma-Aldrich). For equal density, six cortices were used per flask. The astrocytes were cultured at 37 °C and 6% *v*/*v* CO_2_, with a total medium exchange every second day. 

After a cultivation time of 7–8 days, the astrocytes formed a monolayer but still contained unwanted microglia and oligodendrocyte precursor cells. To obtain a pure astrocyte culture, T-75 flasks were initially shaken for 1 h on an orbital shaker (New Brunswick Scientific (by Fisher Scientific GmbH, Schwerte, Germany) at 250 rpm and 37 °C. Then the complete medium was exchanged, and the flasks were shaken overnight. Subsequently, the medium was exchanged and 20 µM cytosine-1-β-d-arabinofuranoside (Ara-C, Sigma Aldrich) was added for 48–72 h. 

For the co-cultivation with hippocampal neurons, pure astrocytes were plated in cell culture inserts (pore size: 0.4 µm; BD Falcon). Therefore, the astrocytes were incubated with trypsin/EDTA (0.1% *w*/*v* trypsin with EDTA in MEM, both from Gibco) for 5–10 min at 37 °C with 6% *v*/*v* CO_2_. Subsequently, 25,000 astrocytes were plated in the inserts pre-coated with 10 µg/mL poly-D-lysine. The main purpose to use wild-type cortical astrocytes in our co-culture system was the prolongation of the cultivation time of hippocampal neurons in completely defined medium, as previously described [29,35]. As we used identical culture conditions for wild-type and the diverse mutant neurons, differences in neuronal biology emerging in our approach should exclusively be caused by the different genotypes of the neurons, that is, the deletion of the corresponding *Vav* genes.

To cultivate hippocampal neurons, pregnant mice were euthanized by cervical dislocation and embryonic mice (E 15.5) were removed. The hippocampi were dissected carefully and relieved from meninges and surrounding tissue. Then, the hippocampi were collected in dissection medium consisting of HBSS (Gibco), 0.6% *v*/*v* glucose (J.T. Baker) and 10 mM HEPES (Gibco). Afterwards, the hippocampal tissue was digested using 30 U Papain (Worthington) in MEM (Gibco) with 40 µg/mL µL DNase and 0.24 mg/mL L-cysteine for 15 min at 37 °C. The digestion solution was replaced by hippocampus medium containing MEM (Gibco), 2% *v*/*v* B27 (Gibco), 0.1% *v*/*v* ovalbumin (Sigma-Aldrich), 10 mM sodium pyruvate (Gibco) and 0.1% *v*/*v* gentamicin (Sigma-Aldrich). After three washing steps, the hippocampi were triturated mechanically to a single cell suspension. To analyse the neuronal morphology, 30,000 cells of each condition were plated out on glass coverslips (Thermo Scientific) pre-coated with 15 µg/mL poly-L-ornithin (Sigma-Aldrich) in 4-well plates (Nunc).

For the indirect co-cultivation with astrocytes and the analysis of synapse formation, it was necessary to cultivate a higher number of neurons. Therefore, 35,000 cells were plated out on glass coverslips, also pre-coated with 15 µg/mL poly-L-ornithine (Sigma-Aldrich), in 24-well plates (BD Falcon). After 1 h, the cell culture inserts (Corning GmbH, Kaiserslautern, Germany) with astrocytes were added to the neurons. In a previous step, the astrocyte medium was replaced by hippocampus medium. The indirect co-cultures were composed of neurons from SV129, *Vav3*^−/−^, *Vav2*^−/−^ and *Vav2*^−/−^/*3*^−/−^ mice and astrocytes from SV129 mice. The cells were cultured under constant conditions at 37 °C and 6% CO_2_ in a humidified incubator.

The number of neurons plated in our culture system is not sufficient to apply commercially available assay kits to measure the activation of RhoA/Rac1/Cdc42 directly. Therefore, we compared different genotypes, namely wild-type, and three mutant (*Vav2^−/−^*, *Vav3^−/−^* and *Vav2^−/−^/3^−/−^*) mouse lines under identical culture conditions. Any difference measured in our assay system should reflect functional differences for neuronal differentiation of the genes under study. 

### 4.4. Immunocytochemistry

In order to analyse the axonal and dendritic parameters, hippocampal neurons were fixed after 3 and 5 DIV. Therefore, the medium was carefully removed and replaced by pre-warmed 4% *w*/*v* paraformaldehyde (Sigma-Aldrich) for 10 min. After three washing steps with PBS, the cells were incubated with the primary antibody solution over night at 4 °C in a humid chamber. Previously, primary antibodies were diluted in PBT1 (PBS with 0.1% *v*/*v* Triton X-100; Sigma-Aldrich and 1% *w*/*v* BSA; AppliChem). Next, the primary antibody solution was aspirated, and the cells were washed three times with PBS/A (PBS containing 0.1% *w*/*v* BSA; AppliChem). Secondary antibodies were diluted in PBS/A and incubated for 1 h at room temperature. After that, cells were rinsed three times with PBS and once with Milli-Q water (Merck Millipore, Darmstadt, Germany). In a final step the cells were covered on microscope slides (Thermo Fisher Scientific, Bleiswijk, The Netherlands) using Immumount (Thermo Scientific) and stored at 4 °C until use.

To examine changes in structural synapses, neurons were co-cultured indirectly with astrocytes and fixed with 4% *w*/*v* paraformaldehyde (Sigma-Aldrich) after 14 and 21 days in culture. After fixation, neurons were rinsed three times with PBS and incubated for 20 min with 25 mM glycine (J.T.Baker). In a following step, the glycine solution was replaced by a blocking buffer consisting of PBS with 10% *v*/*v* horse serum (Gibco) and 0.1% *v*/*v* Triton X-100 (Sigma-Aldrich) for 1 h. Primary antibodies were diluted in blocking buffer and incubated for 1 h in a humid chamber. After washing with blocking buffer twice, the secondary antibodies were also diluted in blocking buffer followed by incubation for 1 h in a dark humid chamber. Finally, the cells were rinsed twice with blocking buffer, once with PBS, once with Milli-Q water (Millipore) and finally covered on microscope slides (Thermo Scientific) with Immumount (Thermo Scientific). The samples were stored at 4 °C until images were taken at the microscope.

### 4.5. Electrophysiology 

The spontaneous network activity of cultured wild-type and *Vav3*^−/−^ neurons was measured with multielectrode arrays (MEA, Multichannel Systems). In a first step the electrode fields were coated with 0.05% *v*/*v* poly-ethylenimine (PEI, Sigma-Aldrich) for 1 h at room temperature. After carefully removing the PEI, the MEAs were washed three times with Milli-Q water (Millipore) and air dried under ultraviolet light. Then, the electrode fields were coated with 5 µg/mL *w*/*v* laminin (Thermo Scientific) for 20 min at 37 °C. Afterwards, the laminin solution was removed and replaced by the neuronal cell suspension. A total number of 30,000 neurons in 30 µL hippocampus medium (1 × 10^6^ cells per ml) was plated out per electrode field. The cell density on electrode fields was visually controlled under the microscope and replenished by addition of another 30 µL of cell suspension, if necessary.

After an incubation time of 10 min, the MEAs were flooded with 1 mL hippocampus medium and stored for 1 h at 37 °C and 6% *v*/*v* CO_2_ to settle and adhere to the substrate. The cell culture inserts (BD Falcon) with astrocytes were added using specially constructed racks after replacing the astrocyte medium with hippocampus medium. Then the co-cultures were transferred into plastic boxes with special covers consisting of fluorinated ethylene-propylene [32]. The cultivation succeeded at 37 °C and 6% *v*/*v* CO_2_ in a humidified incubator.

The measurement of the spontaneous network activity was performed after 14 and 21 DIV. First, the MEAs were positioned on a 35 °C pre-heated pre-amplifier and left for 10 min to avoid an unwanted activity, caused by the transport. Then, the spontaneous network activity was recorded for 10 min using a sample rate of 20 kHz, considering all 60 electrodes. The data was recorded with the program MCRack (Version 3.9.0, Multichannel Systems, Reutlingen, Germany). A high-pass filter with an adjusted frequency of 200 Hz eliminated field potentials from the raw data. In addition, a spike detector was used, which recorded single spontaneous amplitudes 4.5-fold higher than the standard deviation. In order to detect bursts, the following settings were adjusted: maximal interval initiating a burst, 10 ms; maximal interval terminating a burst, 100 ms; maximal interval between two bursts, 210 ms; minimal duration of a burst, 50 ms; minimal number of spikes per burst, 5.

To remove cells, the medium was aspirated, replaced by 1% *w*/*v* tergazyme (Alconox) and incubated overnight at room temperature. After aspirating and discarding, the degraded cells the MEAs were washed three times with Milli-Q water (Millipore) and stored at 4 °C until next usage.

### 4.6. Microscopy 

Images of MAP2/Tau-immunostained neurons were recorded using a fluorescence microscope (AxioPlan 2, Zeiss, Jena, Germany) with an affiliated digital camera (AxioCam MRm, Zeiss). The associated AxioVision 4.5 software (Zeiss) was used for the documentation. Recordings of immunostained synaptic proteins were taken with the confocal laser scanning-microscope LSM 510 Meta (Zeiss). A z-stack width of 0.25 µm was adjusted for the scanning procedure. The number of z-stacks varied between 5 and 7 depending on the size of the individual neuron. Afterward, every single image was assembled to an overlay. The settings for the gain and threshold were kept constant for all experimental repetitions.

### 4.7. Quantification

To compare the morphology of wild-type, *Vav3*^−/−^, *Vav2*^−/−^, and *Vav2*^−/−^*/3*^−/−^ neurons, different axonal and dendritic properties were analysed. The axonal parameters included length and total number of branches. In addition, the number of dendrites per neuron and the length of the longest dendrite were measured. The program ImageJ was used to examine 50–65 neurons per experimental replicate. All data are represented as the increase/decrease relative to the mean of the wild-type condition. Therefore, the following formula was used: $\frac{\left(value-WT\;mean\right)}{WT\;mean}\times 100$.

Quantification of the synaptic puncta was carried out with the Plug-in “puncta analyser” for ImageJ from Barry Wark (licensed under http://www.gnu.org/copyleft). In order to measure the number of presynaptic Bassoon and postsynaptic PSD-95 puncta following settings had been adjusted: ball radius, 50 pixels; size (pixel2), 2-infinity; circularity, 0.00–1.00 [74]. Co-localizations of the fluorescent signals were considered as indications for structural synapses [35]. For the statistics, 20 immunostained neurons were evaluated per experimental procedure. Finally, the numbers of synaptic puncta of wild-type, *Vav3*^−/−^, *Vav2*^−/−^ and *Vav2*^−/−^/*3*^−/−^ neurons were compared with each other and presented as the relative increase/decrease as mentioned before. The experiments with MEAs were performed with wild-type and *Vav3*^−/−^ neurons. To compare the network activity of cultured hippocampal neurons, data of all 60 electrodes were considered [32]. The number of spikes and bursts were quantified for all conditions.

### 4.8. Statistics

For the statistical analysis, the program IBM SPSS Statistic was used. First the Kolmogorov–Smirnov test was applied to identify the distribution of all data sets. Data sets concerning the axonal and dendritic parameters as well as the immunocytochemical synaptic puncta analysis were not normally distributed. For this reason, the Kruskal–Wallis test was chosen to analyse the level of significance. The values of the MEA analysis were also not normally distributed. Here, the Mann–Whitney *U*-test was applied to examine the significance. The detailed number of independent experiments and the corresponding level of significance are indicated in the figure legends. The significance level was set at *p* ≤ 0.05. Data are given with the mean value and the standard error of the mean (±SEM).

## Supplementary Materials

Supplementary materials can be found at https://www.mdpi.com/1422-0067/21/3/856/s1.

## Abbreviations

Ara-C	cytosine-1-β-d-arabinofuranoside
Cdc42	cell division control protein 42
CNF-1	*Escherichia coli* necrotizing factor 1
CNS	central nervous system
DIV	days in vitro
DNase	desoxyribonuclease
E	embryonic
GABA	γ-aminobutric acid
GDP	guanosine diphosphate
GEF	guanine nucleotide exchange factor
GTP	guanosine triphosphate
KO	knockout
LSM	laser scanning microscope
MAP2	microtubule-associated protein 2
MEA	multielectrode array
mRNA	messenger ribonucleic acid
PSD-95	postsynaptic density protein 95
Rac1	Ras related C3 botulinum toxin substrate 1
RhoA	Ras homolog gene family member A
RhoG	Ras homolog gene family member G
SEM	standard error of the mean
WT	wild-type
