# Supplementary material for: Deletion of the Nucleotide Exchange Factor Vav3 Enhances Axonal Complexity and Synapse Formation but Tampers Activity of Hippocampal Neuronal Networks In Vitro
Source: Int J Mol Sci. 2020 Jan 28;21(3):856. doi: 10.3390/ijms21030856 (PMC7037001; doi:10.3390/ijms21030856)
Supplement: Supplementary file 1 [file ijms-21-00856-s001.pdf]

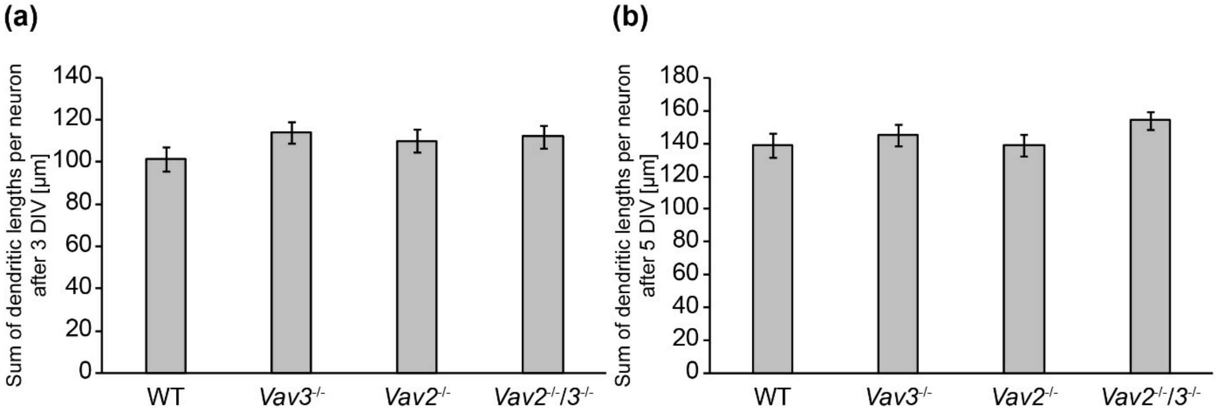

Supplementary figure S1: Analysis of the total sum of dendritic lengths per neuron. (**a**) The sum of all dendrites per neuron was calculated and compared between all groups. No significant differences could be observed after three days of cultivation; (**b**) In addition, the sum of all dendrites per neuron was determined after five days *in vitro*. Here, also no significant alterations could be registered.

Statistics: Four independent experiments (N=4) were performed and the dendrites of 15 neurons (n=5) per experimental condition were measured. Data are shown as mean ± SEM (One-way ANOVA with Tukey's multiple comparison test; p ≤ 0,05)

Supplementary table S1: Absolute values of axonal parameters

Axonal length				
Cultivation time	wild-type	$Vav3^{-/-}$	$Vav2^{-/-}$	$Vav2^{-/-}/3^{-/-}$
DIV3	102.22 ± 2.50 µm	122.57 ± 2.65 µm	113.40 ± 2.66 µm	124.55 ± 3.01 µm
DIV5	164.85 ± 3.70 µm	199.79 ± 4.39 µm	170.23 ± 4.41 µm	204.86 ± 5.51 µm
Axonal branch numbers				
Cultivation time	wild-type	$Vav3^{-/-}$	$Vav2^{-/-}$	$Vav2^{-/-}/3^{-/-}$
DIV3	5.87 ± 0.26	8.40 ± 0.36	6.21 ± 0.28	6.82 ± 0.32
DIV5	10.47 ± 0.36	14.02 ± 0.46	11.31 ± 0.48	13.51 ± 0.47

Supplementary table S2: Absolute values of dendritic parameters

Number of primary dendrites				
Cultivation time	wild-type	$Vav3^{-/-}$	$Vav2^{-/-}$	$Vav2^{-/-}/3^{-/-}$
DIV3	4.81 ± 0.12	4.96 ± 0.12	4.81 ± 0.11	5.07 ± 0.13
DIV5	5.86 ± 0.13	5.78 ± 0.13	4.66 ± 0.13	5.66 ± 0.12
Length of the longest dendrite				
Cultivation time	wild-type	$Vav3^{-/-}$	$Vav2^{-/-}$	$Vav2^{-/-}/3^{-/-}$
DIV3	32.49 ± 0.80 µm	40.66 ± 0.93 µm	37.63 ± 0.85 µm	43.26 ± 0.97 µm
DIV5	40.32 ± 0.92 µm	50.51 ± 1.44 µm	41.19 ± 1.00 µm	49,87 ± 1.13 µm
Sum of dendritic lengths per neuron				
Cultivation time	wild-type	$Vav3^{-/-}$	$Vav2^{-/-}$	$Vav2^{-/-}/3^{-/-}$
DIV3	101,46 ± 5.51 µm	113,87 ± 5.10 µm	110,08 ± 5.41 µm	111,98 ± 5.64 µm
DIV5	139,03 ± 7.30 µm	145,36 ± 6.48 µm	138,97 ± 6.27 µm	154,05 ± 5.50 µm

Supplementary table S3: Absolute values of synaptic puncta analysis

Number of Bassoon puncta				
Cultivation time	wild-type	Vav3$^{-/-}$	Vav2$^{-/-}$	Vav2$^{-/-}$/3$^{-/-}$
DIV14	2953.47 ± 118.69	3850.26 ± 159.56	3440.07 ± 144.61	3816.90 ± 150.67
DIV21	5089.91 ± 179.69	5241.44 ± 212.81	4873.95 ± 246.95	5658.81 ± 190.41
Number of PSD-95 puncta				
Cultivation time	wild-type	Vav3$^{-/-}$	Vav2$^{-/-}$	Vav2$^{-/-}$/3$^{-/-}$
DIV14	3979.59 ± 130.80	4722.29 ± 199.04	4592.82 ± 185.23	5160.84 ± 227.02
DIV21	6625.03 ± 231.25	5686.12 ± 223.01	6226.35 ± 355.10	7099.78 ± 274.39
Number of colocalized puncta				
Cultivation time	wild-type	Vav3$^{-/-}$	Vav2$^{-/-}$	Vav2$^{-/-}$/3$^{-/-}$
DIV14	1501.23 ± 59.02	1935.94 ± 78.11	1676.16 ± 69.28	1942.23 ± 80.02
DIV21	3075.82 ± 112.24	2713.87 ± 109.62	3158.68 ± 152.82	3021.62 ± 123.01
